# Prevalence and associated factors of sexual, psychological, and physical violence among physical therapists in their clinical role in Spain: a national web-based cross-sectional survey

**DOI:** 10.1093/joccuh/uiae013

**Published:** 2024-04-11

**Authors:** Tania Boo-Mallo, Manuel Oviedo-de-la-Fuente, Alicia Martínez-Rodríguez

**Affiliations:** Universidade da Coruña (University of A Coruña), Department of Physiotherapy and Biomedical Science, Psychosocial Intervention and Functional Rehabilitation Group, Oza, 15071 A Coruña, Spain; Universidade da Coruña (University of A Coruña), CITIC, Department of Mathematics, Elvina 15071 A Coruña, Spain; Universidade da Coruña (University of A Coruña), Department of Physiotherapy and Biomedical Science, Psychosocial Intervention and Functional Rehabilitation Group, Oza, 15071 A Coruña, Spain

**Keywords:** workplace violence, prevalence, physical therapy specialty, risk factors, sexual harassment, physical abuse

## Abstract

**Objectives:**

To determine the extent of career-long and 12-month exposure to sexual, physical, and psychological/verbal violence committed by patients or their companions among physical therapists in Spain. Additionally, to identify the factors associated with such exposure.

**Methods:**

This study employed an observational cross-sectional approach. Initially, a questionnaire was developed and validated using a convenience sample. Subsequently, it was distributed via email to all physical therapists registered in Spain in the first quarter of 2022. Individual risk models were created for each type of violence experienced within the past 12 months.

**Results:**

The prevalence of violence encountered by physical therapists throughout their careers was 47.9% for sexual violence, 42.7% for psychological/verbal abuse, and 17.6% for physical abuse. Lower values were observed within the last 12 months (13.4%, 15.8%, and 5.2%, respectively). Statistical risk modeling for each type of violence experienced in the past 12 months indicated that the common precipitating factor for all forms of violence was working with patients with cognitive impairment. Working part-time appeared to be a protective factor. Other factors, such as the practitioners’ gender, practice setting, or clinic location showed variations among the diverse types of violence.

**Conclusions:**

The exposure to type II workplace violence within the last 12 months among physical therapists in Spain (Europe) is not so high as in some other world regions. Various individual, clinical, and professional/organizational risk factors have been identified in connection with type II workplace violence. Further research is warranted to compare the violence experienced once the COVID pandemic has subsided.

## Key points

- **What is already known on this topic:** It is known that health care workers are more likely to suffer violence than other workers, although differences have been found between health professions, practice settings, and cultures. However, the COVID-19 pandemic has conditioned the clinical relationship with patients, which may have had an impact on the occurrence of violence. In addition, this phenomenon has not been extensively studied in physical therapy, which has specific conditioning factors. Physical therapy involves physical contact and sometimes the experience of pain, which can be misinterpreted by the patient and serve as a pretext to justify different types of violence.
- **What this study adds:** This is the first national-level study of type II workplace violence prevalence—sexual, physical, and psychological/verbal—against Spanish physical therapists and factors associated with each form violence. The current data highlight the importance of sexual and psychological/verbal violence, with higher reported rates than in the case of physical violence. Barriers and facilitators identified in previous studies persisted, yet we also identified new personal and organizational risk and protective factors.
- **How this study might affect research, practice, or policy:**The findings of this study, relevant to both practitioners and policy-makers, may be used to improve safety in the clinical setting. Further research is needed to better understand the underlying causes of the identified barriers and facilitators.

## 1. Introduction

The World Health Organization defines workplace violence (WPV) as “intentional use of physical force or power, threatened or actual, against oneself, another person or against a group or community that either results in, or has a high likelihood of resulting in, injury, death, psychological harm, incorrect development or deprivation.”^1^ Therefore, this definition encompasses various forms of violence, such as physical, verbal, psychological (eg, intimidation, threats of harm), and sexual behaviors.^2^ Sexual violence (SV) is also known as inappropriate sexual behavior, referring to any physical or verbal act involving an explicit or perceived sexual content that is deemed unacceptable within the social context in which it occurs.^3^

WPV is a significant issue within the health sector, and is of even greater concern compared with other sectors.^2^ Health workers experience assaults at a rate four times higher than those in other professions.^4^ When the perpetrator and the worker have a client/provider relationship, it is classified as type II occupational violence.^5^ Most of the WPV incidents in health care are of type II and can be attributed to patients and their families/visitors,^6^ especially in clinical settings.^7^

The global extent of WPV must be addressed, in addition to recognizing its risk factors. This knowledge is crucial to enable at-risk professionals to take preventive measures and safeguard their health and well-being.^8^

According to a systematic review, 61.9% of health care workers worldwide have been exposed to some form of WPV in the past year, with 24.4% experiencing physical violence (PV), and 42.4% encountering nonphysical violence, including sexual harassment (12.4%).^9^ Variations in the type and prevalence of WPV exposure were found among countries, practice settings, and health care professions.^10^ Nurses had the highest exposure to any form of WPV, followed by physicians.^9^ However, the medical staff were more likely to experience PV, whereas nurses faced a higher risk of sexual harassment.^10^ Although there is limited research on physical therapists, one study conducted in the United States found that the prevalence of SV over their careers reached as high as 84%, with 47% experiencing it in the past 12 months.^11^ Another study involving UK physical therapists, especially those working in mental health settings, reported a career-long prevalence of PV exceeding 50% and a 24% prevalence in the last 12 months.^12^ It is important to note that Spain’s culture, characterized by close physical contact, likely affects how violence is perceived in contrast to northern countries.^13^ Initial research showed that 11% of Spanish public health workers had experienced physical aggression, whereas 64% had encountered nonphysical type II WPV in the past year.^14^ Notably, the victims in 77% of these reported events were women.^15^ However, specific data for physical therapists in both public and private practices, where most of Spanish physical therapists work, remain undisclosed. Physical therapists play a fundamental role in close physical contact with patients,^16^ even when patients are in pain,^12^ which can make them more vulnerable to violence, given the nature of their work.^17^ Additionally, physical therapists use techniques in intimate body areas, which may be misinterpreted^18^ and associated with an increased risk of SV.^19^

The prevalence of type II WPV among Spanish physical therapists and the factors associated with the various forms of aggression remain largely unexplored. Consequently, this study aimed to achieve 2 primary objectives: (1) to assess the frequency and characteristics of type II WPV experienced by physical therapists in Spain, with a focus on sex differences, and (2) to identify the personal, professional, and clinical factors linked to this phenomenon.

## 2. Material and methods

This observational cross-sectional study adhered to the STROBE statement. It received approval from the Ethical Committee of the State University of A Coruña (Spain) with the code CEID-UDC 2021-005-2, in accordance with the principles outlined in the Helsinki Declaration.

### 2.1. Survey development

The development of the questionnaire occurred in 3 phases. First, questions from relevant papers identified in a literature review were adapted,^18^^,^^20^^,^^21^ especially from a study focused on physical therapists.^11^ Next, the primary items were refined with input from a panel of 6 physiotherapists responsible for the Commission for Monitoring of Violence within the General Council of Colleges of Physiotherapists of Spain (GCCPS), as well as those associated with the “Spanish MeToo Physio Movement.” Finally, the questionnaire’s comprehensibility and the time required to complete it were assessed with a convenience sample of 14 physical therapists (21% men, 79% women). This pilot-test prompted adjustments, primarily in 2 response options, specifically those related to the age of the aggressor and working alone. This change involved increasing the number of response categories.

The questionnaire included inquiries about the demographic and professional background of the respondents, such as sex, age, and clinical experience treating patients.^22^ Additionally, it encompassed questions regarding the characterization of violent incidents categorized into 3 types: SV, PV, and psychological/verbal violence (PVV). The majority of the survey consisted of closed-response questions, with the exception of 3 questions that were open-ended.

The questionnaire collected data for 2 periods: the respondent’s career and last 12 months. It is important to note that only physical therapists who had worked for a minimum of 3 of the last 12 months were eligible to answer questions related to this period. The duration was established as the minimum exposure time.

Finally, the GCCPS conducted an electronic survey that was distributed via email to its members across the country. The survey was available for 2 months, from January 10 to March 14, 2022. Email reminders were sent every 3 weeks to encourage participation. Respondents were explicitly informed that participation was entirely voluntary and anonymous, and they were required to provide informed consent to access the survey. Data confidentiality was ensured through the use of Microsoft Forms software (Microsoft Office, Microsoft Corporation, USA) pursuant to an agreement with the University of A Coruña. Eligible participants for this study were physical therapists in Spain who had experience in direct patient care.

### 2.2. Data analysis

The data were analyzed using R statistical software (version 4.2.2) with stepAIC function of the MASS package and gam function of the mgcv package. For continuous variables, mean and SD were computed. For categorical variables, absolute and relative frequencies (percentages) were calculated. We applied the χ^2^ test or Fisher exact test, depending on the expected frequencies, with a significance level of α = .05.

In the bivariate analysis, we considered several variables, including sex, sexual orientation, training in WPV, clinic location, working hours (part-time/full-time), ownership of the center, patient access profile, practice setting, treatment in private spaces, working with patients with cognitive impairment (PWCI), and working alone.

For the analysis of different types of violence (SV, PV, and PVV), we employed various additive (nonlinear) logistic regression models. To mitigate potential biases, we ensured that the study cohort consisted of professionals with a minimum of 3 months of clinical experience within the last 12 months. Two weighting schemes were considered: one with equiprobable weights (GAM model) and the other with weights inversely proportional to class frequencies (GAM.W model), which was found to be more suitable for imbalanced class distributions. In terms of variable selection, we included all the variables from the bivariate analysis, except for the working area variable (excluded due to the high number of categories and insufficient observations in some of them), allowing the model to select the most relevant variables of professional interest independent of bivariate significance.^23^ The best-fitting model was determined using a stepwise Akaike corrected information criterion. At each step of this process, potential covariates eligible for inclusion or exclusion in the model were selected through a forward/backward stepwise logistic regression procedure using only the variables that showed significance for each type of violence. The GAM model was adjusted by estimating a linear effect for categorical variables and a nonlinear effect for numerical covariates (age and clinical experience). This variable selection strategy was chosen for its interpretability, simplicity, and to avoid overfitting and collinearity.^24^

Atypical observations, often referred to as outliers, were identified in the dataset, including individuals aged ≥60 years or with over 30 years of clinical experience, representing 1.5% of the sample. These outliers were filtered to create a less heterogeneous sample, reduce bias in parameter estimates, and obtain a more stable and robust GAM model.

We evaluated all models using the following metrics: sensitivity, specificity, AUC (area under the curve), and pseudo-R^2^. It is worth noting that accuracy is not a suitable metric for unbalanced datasets. We adhered to best coding practices to ensure the reproducibility and replicability of our results.

## 3. Results

The number of respondents accepting the informed consent while also having had a history of treating patients throughout their careers totaled 2942. However, 91 of them had not worked for at least 3 of the last 12 months, and, therefore, they were directed to answer more general questions related to violence experiences throughout their careers. An additional 9 responses were excluded from the analysis because the individuals identified their sex as “other,” and the group size was too small for meaningful comparisons (6 responses) or due to inconsistencies in their responses (3 responses). Seventeen responses were also disregarded due to incorrectly recorded data regarding clinical experience, but only for this specific item.

In summary, the majority of the respondents were women (72.8%), held full-time jobs (78.8%), and worked in private health care settings (76%). The age of respondents ranged from 23 to 74 years, with a mean age of 37.27 years (SD 8.33). The number of years spent in direct patient care varied from 3 months (0.25 years) to 49 years, with an average of 12.77 years (SD 7.94). Additional detailed information, including a breakdown by sex, is shown in Table 1.

**Table 1:** The characteristics of respondents in the population who worked for at least 3 months in the last 12 months^a^

**Characteristics**	**Total**	**Women (*n* = 2068)**	**Men (*n* = 774)**	***P*** ^**b**^
**Age, mean (SD), y**	37.27(SD 8.34)	37.07(SD 8.05)	37.82(SD 9.03)	
**Clinical experience, mean (SD), y**	12.77(SD 7.94)	12.82(SD 7.78)	12.62(SD 8.35)	
**Clinic location,** ^**c**^ ***n* (%)**				<.001*
**>10 000 inhabitants**	2329 (81.9)	1662 (80.4)	667 (86.2)	
**≤10 000 inhabitants**	504 (17.7)	400 (19.3)	104 (13.4)	
**Sexual orientation, *n* (%)**				.5
**Heterosexual**	2498 (87.9)	1827 (88.3)	671 (86.7)	
**LGBQ+**	175 (6.2)	124 (6)	51 (6.6)	
**No answer**	169 (5.9)	117 (5.7)	52 (7.1)	
**Training in workplace violence, *n* (%)**				.926
**Yes**	357 (12.6)	261 (12.6)	96 (12.4)	
**No**	2485 (87.4)	1807 (87.4)	678 (87.6)	
**Working hours, *n* (%)**				**.002**
**Full-time**	2237 (78.7)	1597 (77.2)	640 (82.7)	
**Part-time**	605 (21.3)	471 (22.8)	134 (17.3)	
**Ownership of the center, *n* (%)**				**.003**
**Private**	2170 (76.4)	1548 (74.9)	622 (80.4)	
**Public**	672 (23.6)	520 (25.1)	152 (19.6)	
**Patient access, *n* (%)**				**<.001**
**No direct payment per session**	1159 (40.8)	889 (43)	270 (34.9)	
**Direct payment per session**	1474 (51.9)	1034 (50.0)	440 (56.8)	
**Private insurance**	209 (7.4)	145 (7.0)	64 (8.3)	
**Practice setting,** ^**d**^ ***n* (%)**				**<.001**
**Inpatient**	328 (11.5)	250 (12.1)	78 (10.1)	
**Outpatient**	533 (18.8)	398 (19.2)	135 (17.4)	
**Health center**	186 (6.5)	146 (7.1)	40 (5.2)	
**Private clinic**	1482 (52.1)	1043 (50.4)	439 (56.7)	
**Patient associations**	68 (2.4)	58 (2.8)	10 (1.3)	
**Home care**	91 (3.2)	59 (2.9)	32 (4.1)	
**Academic or research institution**	6 (0.2)	3 (0.1)	3 (0.4)	
**School system**	62 (2.2)	49 (2.4)	13 (1.7)	
**Other**	86 (3.0)	62 (3)	24 (3.1)	
**Work area,** ^**d**^ ***n* (%)**				**<.001**
**Cardiopulmonary**	42 (1.5)	31 (1.5)	11 (1.4)	
**Trauma/sports/rheumatology**	1690 (59.5)	1157 (55.9)	533 (68.9)	
**Urogynecology**	74 (2.6)	74 (3.6)	-	
**Neurology**	206 (7.2)	162 (7.8)	44 (5.7)	
**Pediatrics**	123 (4.3)	106 (5.1)	17 (2.2)	
**Geriatrics**	201 (7.1)	155 (7.5)	46 (5.9)	
**Oncology**	7 (0.2)	7 (0.3)	-	
**Aesthetics**	1 (0.0)	1 (0.0)	-	
**Other**	498 (7.5)	375 (18.1)	123 (15.9)	
**Treatment in private spaces, *n* (%)**				.074
**Same on both sides**	642 (22.6)	472 (22.8)	170 (22)	
**More in open areas/gym**	619 (21.8)	470 (22.7)	149 (19.3)	
**More in closed areas (room, cabin)**	1581 (55.6)	1126 (54.4)	455 (58.8)	
**Working alone, *n* (%)**				**.008**
**Yes**	1785 (62.8)	1268 (61.3)	517 (66.8)	
**No**	1057 (37.2)	800 (38.7)	257 (33.2)	
**Working with chaperone, *n* (%)**				**<.001**
**Yes (men)**	110 (3.9)	99 (4.8)	11 (1.4)	
**Yes (woman)**	103 (3.6)	74 (3.6)	29 (3.7)	
**No**	2629 (92.5)	1895 (91.6)	734 (94.8)	
**Working with patients with cognitive impairments, *n* (%)**				.954
**Yes**	1418 (49.9)	1033 (50)	385 (49.7)	
**No**	1424 (50.1)	1035 (50.0)	389 (50.3)	

a Percentages may not sum exactly to 100% due to rounding.

b Values in bold indicate statistically significant results.

c Nonmandatory question.

d Fisher exact test is used when there is at least 1 cell in the contingency table of the expected frequencies below 5.

Abbreviations: LGBQ+, lesbian, gay, bisexual, queer, and other diverse sexual orientations and gender identities.

### 3.1. Prevalence

The results showed that 67.5% of physical therapists had experienced at least 1 type of violence during their professional career, with 27.7% reporting such experiences in the last 12 months.

More specifically, the prevalence of SV throughout their careers, regardless of sex, was 47.9%, whereas PV was reported by 17.6% of participants, and PVV was experienced by 42.7%. In the last 12 months, these figures decreased to 13.4%, 5.2%, and 15.8%, respectively.

Table 2 presents the prevalence of each type of violence for both time periods, categorized by sex. The table indicates that women had a higher prevalence of violence than men in all forms of violence except for PV in the last 12 months. It is worth noting that being a woman was significantly associated with higher rates of general violence and SV in the last 12 months (*P* < .001 in both cases).

**Table 2:** Career and 12-month prevalence of violence by sex^a^

	**Career prevalence**			**12-month prevalence**		
	**Men, % (*n*)**	**Women, % (*n*)**	***P*** ^**b**^	**Men, % (*n*)**	**Women, % (*n*)**	***P*** ^**b**^
**Sexual violence**	23.5 (186)	56.9 (1219)	**<.001**	5.9 (46)	16.2 (334)	**<.001**
**Physical violence**	16.2 (128)	18.1 (387)	.248	6.1 (47)	4.8 (100)	.219
**Psychological/verbal violence**	37.9 (300)	44.5 (952)	**.001**	15.6 (121)	15.8 (327)	.953
**General violence (1 at least or more)**	52.4 (415)	73.1 (1566)	**<.001**	22.7 (176)	29.5 (610)	**<.001**

a Percentages were calculated by number of women or men suffering violence (sexual, physical, or psycho/verbal) by the whole number of women or the whole number of men exposed in each period, respectively.

b Values in bold indicate statistically significant results.

### 3.2. Frequency of experiencing violence

The most common frequency of experiencing any type of violence, for both men and women, was “on more than one occasion and less than once a month.” More women than men reported experiencing SV or PV at least once a month or more. However, none of the types of violence showed statistically significant differences in frequency based on sex (SV, *P* = .280; PV, *P* = .287; PVV, *P* = .923). Additional detailed data can be found in Table 3.

**Table 3:** Frequency of violence by sex suffered in the last 12 months^a^

	**Sexual violence, % (*n*)**		**Physical violence, % (*n*)**		**Psychological/verbal violence, % (*n*)**	
	**Men**	**Women**	**Men**	**Women**	**Men**	**Women**
**Single occasion**	21.7 (10)	20.1 (67)	21.3 (10)	23.0 (23)	24.0 (29)	25.7 (84)
**On more than 1 occasion and less than once a month**	56.5 (26)	46.7 (156)	51.1 (24)	38.0 (38)	46.3 (56)	45.9 (150)
**At least once a month (or equivalent) or more**	21.7 (10)	33.2 (111)	27.7 (13)	39.0 (39)	29.8 (36)	28.4 (93)
***P* value from χ** ^**2**^ **tests**	.280		.287		.923	

a Percentages may not be exactly 100% due to rounding. Percentages by sex were calculated by the whole number of women/men suffering sexual violence, physical violence, and psychological and/or verbal violence.

### 3.3. Experience and risk factors

In the bivariate analysis, statistically significant relationships (*P* < .01) were exclusively observed between the 3 forms of violence studied and patient access, practice setting, treatment in private spaces, and working with PWCI. For more information on the other variables and their impact on the 3 types of violence, refer to Table 4.

**Table 4:** Bivariate analysis between each violence and the physical therapist/job characteristics^a^

	**Sexual violence**			**Physical violence**			**Psychological/verbal violence**		
	**No, *n* (%)**	**Yes, *n* (%)**	***P*** ^**b**^	**No, *n* (%)**	**Yes, *n* (%)**	***P*** ^**b**^	**No, *n* (%)**	**Yes, *n* (%)**	***P*** ^**b**^
**Clinic location**			1.000			.403			**.003**
**>10 000 inhabitants**	1827 (81.9)	287 (82.0)		2006 (82.0)	108 (78.8)		1759 (80.9)	355 (87.2)	
**≤10 000 inhabitants**	405 (18.1)	63 (18.0)		439 (18)	29 (21.2)		416 (19.1)	52 (12.8)	
**Sex**			**<.001**			.137			.868
**Women**	1578 (70.7)	307 (87.7)		1793 (73.3)	92 (67.2)		1586 (72.9)	299 (73.5)	
**Men**	654 (29.3)	43 (12.3)		652 (26.7)	45 (32.8)		589 (27.1)	108 (26.5)	
**Sexual orientation**			**<.001**			.614			.228
**Heterosexual**	2100 (94.1)	311 (88.9)		2285 (93.5)	126 (92)		2037 (93.7)	374 (91.9)	
**LGBQ+**	132 (5.9)	39 (11.1)		160 (6.5)	11 (8.0)		138 (6.3)	33 (8.1)	
**Training in WPV**			.329			**<.001**			**<.001**
**Yes**	274 (12.3)	36 (10.3)		280 (11.5)	30 (21.9)		224 (10.3)	86 (21.1)	
**No**	1958 (87.7)	314 (89.7)		2165 (88.5)	107 (78.1)		1951 (89.7)	321 (78.9)	
**Working hours**			.918			1.000			.066
**Full-time**	1751 (78.4)	276 (78.9)		1919 (78.5)	108 (78.8)		1693 (77.8)	334 (82.1)	
**Part-time**	481 (21.6)	74 (21.1)		526 (21.5)	29 (21.2)		482 (22.2)	73 (17.9)	
**Ownership of the center**			**<.001**			.400			**<.001**
**Private**	1687 (75.6)	302 (86.3)		1888 (77.2)	101 (73.7)		1709 (78.6)	280 (68.8)	
**Public (State)**	545 (24.4)	48 (13.7)		557 (22.8)	36 (26.3)		466 (21.4)	127 (31.2)	
**Patient access**			**.005**			**<.001**			**<.001**
**No direct payment per session**	917 (41.1)	123 (35.1)		951 (38.9)	89 (65)		799 (36.7)	241 (59.2)	
**Direct payment per session**	1163 (52.1)	188 (53.7)		1313 (53.7)	38 (27.7)		1232 (56.6)	119 (29.2)	
**Private insurance**	152 (6.8)	39 (11.1)		181 (7.4)	10 (7.3)		144 (6.6)	47 (11.5)	
**Practice setting**			**.002**			**<.001**			**<.001**
**Inpatient**	250 (11.2)	43 (12.3)		241 (9.9)	52 (38)		220 (10.1)	73 (17.9)	
**Outpatient**	421 (18.9)	66 (18.9)		463 (18.9)	24 (17.5)		382 (17.6)	105 (25.8)	
**Health center**	157 (7.0)	7 (2.0)		160 (6.5)	4 (2.9)		118 (5.4)	46 (11.3)	
**Private clinic**	1155 (51.7)	204 (58.3)		1330 (54.4)	29 (21.2)		1217 (56)	142 (34.9)	
**Other** ^**c**^	249 (11.2)	30 (8.6)		251 (10.3)	28 (20.4)		238 (10.9)	41 (10.1)	
**Treatment in private spaces**			**.001**			**<.001**			**<.001**
**Same on both sides**	496 (22.2)	90 (25.7)		536 (21.9)	50 (36.5)		469 (21.6)	117 (28.7)	
**More in open areas/gym**	500 (22.4)	48 (13.7)		503 (20.6)	45 (32.8)		419 (19.3)	129 (31.7)	
**More in closed areas (room/cabin)**	1236 (55.4)	212 (60.6)		1406 (57.5)	42 (30.7)		1287 (59.2)	161 (39.6)	
**Working with PWCI**			**.001**			**<.001**			**<.001**
**Yes**	1077 (48.3)	202 (57.7)		1161 (47.5)	118 (86.1)		994 (45.7)	285 (70.0)	
**No**	1155 (51.7)	148 (42.3)		1284 (52.5)	19 (13.9)		1181 (54.3)	122 (30)	
**Working alone**			.148			**.027**			**<.001**
**Yes**	1399 (62.7)	234 (66.9)		1559 (63.8)	74 (54.0)		1411 (64.9)	222 (54.5)	
**No**	833 (37.3)	116 (33.1)		886 (36.2)	63 (46)		764 (35.1)	185 (45.5)	

a To obtain a more homogeneous sample, individuals <60 years old or with less than 30 years of clinical experience were selected. Total number of 2582.

b Values in bold indicate statistically significant results.

c Including: school system, academic or research institution, home care, patient associations.

Abbreviations: LGBQ+, lesbian, gay, bisexual, queer, and other diverse sexual orientations and gender identities; PWCI, patient with cognitive impairment; WPV, workplace violence.

**Figure 1:**
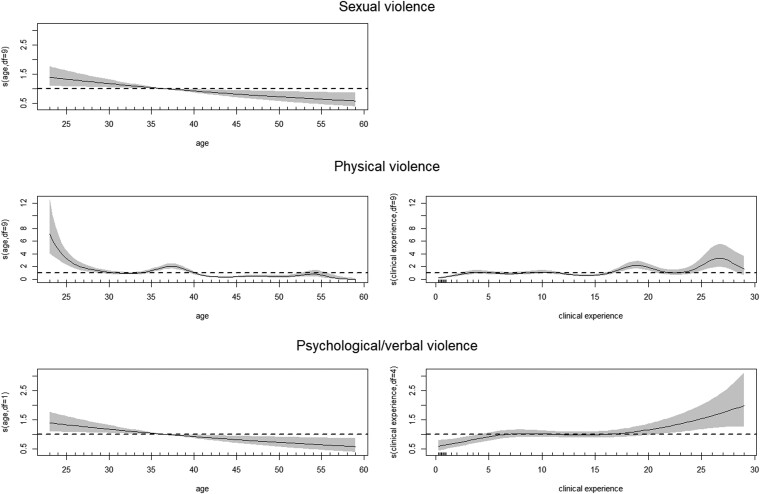
Age and clinical experience effects, considered in a nonlinear model, according to sexual, physical, and psychological/verbal violence, respectively. Clinical experience was not found to be statistically significant in sexual violence. s, spline interpolation.

The results of the multiple logistic regression for categorical covariates can be found in Table 5, whereas the numerical covariates are presented graphically (Figure 1).

**Table 5:** Multivariate analysis to determine risk factors associated with sexual violence, physical violence, and psychological and/or verbal violence

	**Variables (reference)**	**Category**	**Coefficient**	**Odds ratio (95% CI)**	***P*** ^a^
	Sex (women)	Men	−1.22	0.3 (0.25-0.35)	**<.001**
	Sexual orientation (heterosexual)	LGBQ+	0.39	1.48 (1.18-1.86)	**.001**
	Working hours (full-time)	Part-time	−0.35	0.71 (0.61-0.82)	**<.001**
	Patient access (direct payment per session)	No direct payment per session	0.29	1.33 (1.06-1.67)	**.012**
		Private insurance	0.58	1.78 (1.38-2.29)	**<.001**
	Practice setting (health center)	Inpatient	1.08	2.95 (2-4.36)	**<.001**
		Other	0.62	1.86 (1.24-2.79)	**.003**
		Outpatient	0.99	2.69 (1.85-3.92)	**<.001**
		Private clinic	1.27	3.57 (2.37-5.39)	**<.001**
	Treatment in private or open spaces (both)	Open	−0.63	0.53 (0.44-0.65)	**<.001**
		Closed	0.04	1.05 (0.88-1.24)	.611
	PWCI (no)	Yes	0.59	1.80 (1.57-2.06)	**<.001**
**Physical violence**	Sex (women)	Men	0.37	1.44 (1.24-1.67)	**<.001**
	Clinic location (>10 000 inhabitants)	≤10 000	0.51	1.66 (1.40-1.97)	**<.001**
	Training in WPV (no)	Yes	0.72	2.05 (1.70-2.48)	**<.001**
	Working hours (full-time)	Part-time	−0.28	0.76 (0.64-0.90)	**.002**
	Ownership of the center	State	−0.79	0.46 (0.37-0.56)	**<.001**
	Patient access 7	No direct payment per session	0.59	1.81 (1.43-2.3)	**<.001**
		Private insurance	−0.14	0.87 (0.64-1.19)	.387
	Practice setting (health center)	Inpatient	1.73	5.62 (3.91-8.09)	**<.001**
		Other	1.08	2.94 (2.03-4.24)	**<.001**
		Outpatient	0.15	1.17 (0.82-1.66)	.399
		Private clinic	−0.04	0.96 (0.64-1.43)	.841
	Treatment in private or open spaces (both)	Open	−0.59	0.55 (0.46-0.67)	**<.001**
		Closed	−0.68	0.51 (0.43-0.60)	**<.001**
	PWCI (no)	Yes	1.34	3.82 (3.25-4.48)	**<.001**
**Psychological/verbal violence**	Clinic location (>10 000 inhabitants)	≤10 000	−0.42	0.66 (0.56-0.78)	**<.001**
	Training in WPV (no)	Yes	0.54	1.72 (1.45-2.04)	**<.001**
	Working hours (full-time)	Part-time	−0.23	0.8 (0.68-0.93)	**.003**
	Ownership of the center	State	−0.56	0.57 (0.47-0.69)	**<.001**
	Patient access (direct payment per session)	No direct payment per session	1.08	2.95 (2.38-3.65)	**<.001**
		Private insurance	1.19	3.29 (2.58-4.19)	**<.001**
	Practice setting (health center)	Inpatient	−0.55	0.58 (0.44-0.76)	**<.001**
		Other	−0.94	0.39 (0.29-0.52)	**<.001**
		Outpatient	−0.59	0.55 (0.43-0.72)	**<.001**
		Private clinic	−0.54	0.58 (0.43-0.8)	**.001**
	PWCI (no)	Yes	0.87	2.38 (2.09-2.70)	**<.001**

a Values in bold indicate statistically significant results.

Abbreviations: LGBQ+, lesbian, gay, bisexual, queer, and other diverse sexual orientations and gender identities; PWCI, patient with cognitive impairment; WPV, workplace violence.

Regarding the first set of variables, part-time work was associated with a 30% decrease in the odds of experiencing SV, a 25% decrease in PV, and a 20% decrease in PVV. On the other hand, those regularly working with PWCI had significantly increased odds of reporting any form of violence, with 1.8 times higher odds for SV, 3.81 times for PV, and 2.37 times for PVV. Other contributing factors to violence rates included the practice setting and patient access profile, where direct payment per session generally functioned as a protective factor against all types of violence. Sex and treatment in private spaces were statistically significant in SV and PV models, whereas training in WPV, clinic location, and center ownership were significant for PV and PVV. Sexual orientation was found to be relevant only in the context of SV.

For these 2 continuous variables, age and clinical experience, the estimates for the best model (GAM.W) can be found in Figure 1. In SV, age displayed a decreasing trend in younger age groups and an increasing trend in older age groups, forming a V-shaped pattern. This indicates a protective effect in the age range of 30 to 56. However, age and clinical experience did not exhibit a clear trend in their effect estimation in PV and PVV. It is important to consider these results together, as these variables exhibit an inverse relationship in their effect estimation, showing a high correlation of 95%.

Table S1 presents sensitivity ranging from 67.8% to 76.6%, and specificity ranging from 64.6% to 73.9%. As per the pseudo-*R*^2^ values, the models explain 11.2% of the variability in PVV, 16.2% in SV, and 22.8% in PV. The best results were obtained with the GAM.W model.

## 4. Discussion

To the best of our knowledge, this study represents the first national-based examination of violence against Spanish physical therapists in their clinical roles. It takes into account various aspects of violence, including sexual, physical, and psychological/verbal, while also analyzing the personal, professional, and clinical factors associated with each of these forms of violence. Furthermore, this study stands out as the largest of its kind, made possible through the support of the GCCPS.

The prevalence of type II violence varies considerably, ranging from 9.5% to 74.6% in outpatient physician clinics, with a focus on the previous 12 months.^25^ The findings of our current research, which reveal a global prevalence of 27.7% in the last 12 months, align with existing literature but fall within the lower range. This is especially notable in the case of SV when compared with US physical therapists (84% for career and 47% for the last 12 months).^11^

Several factors may help explain the lower rates observed in our study. Firstly, the presence of the COVID-19 pandemic has had a significant impact on physical therapy practice in 2020, particularly in private practice,^26^ and rehabilitation services remained disrupted in 2021.^27^ As a result, fewer physical therapists were likely working in private practice, and our data suggest that they are more likely to experience SV but less likely to experience PV and PVV. Consequently, a more pronounced decrease in SV rates compared with PV and PVV rates is expected.

Secondly, methodological and cultural factors can influence violence rates. In this research, we specifically focused on type II WPV, that is, violence originating from patients, companions, or relatives. However, some studies have identified physicians^28^ and colleagues as significant perpetrators,^29^^,^^30^ a consideration not addressed in this study. Regarding cultural influences, studies have indicated that Asian countries, North America, and Australasia tend to have higher rates of WPV than Europe.^9^ These differences may be attributed, in part, to variations in social relational factors, such as cultural contact patterns^13^ and health care workers’ confidence in reporting violent incidents.^9^ Additional differences may be rooted in health care system funding, the supply of services, government health expenditures,^31^ the number of health professionals,^32^ and remuneration practices for health workers. These factors can result in heavier workloads, diminished trust in health care professionals, and a compromise in patient-centered care, thereby facilitating higher levels of violence exposure.^33^ Spain’s health care system is characterized by public expenditure, universal health coverage, strong primary care, and positive outcomes in terms of equality, health, and life expectancy.^34^ Consequently, the lower rates observed in Spain compared with the United States are in line with these findings.^9^

Regarding personal factors, practitioners’ sex, sexual orientation, and age have been connected with WPV. Women were found to be more susceptible to SV, whereas men had a higher likelihood of experiencing PV. These findings are consistent with previous studies.^9^^,^^11^^,^^35^ The sexualization of women and their perceived caregiver profile contribute to higher rates of SV against women.^36^ Additionally, nonheterosexual or noncisgender physical therapists were also more likely to be victims of SV, aligning with findings related to sexual and gender minorities in the general population who face disproportionate levels of violence.^37^ Finally, age may also play a significant role, as younger physical therapists have been associated with a higher risk of experiencing any type of violence.^9^^,^^25^ In the current study, we observed a nonlinear relationship between age or clinical experience and violence. Further research is needed to clarify the relationship between these variables and identify groups at a higher risk of experiencing violence.

At a professional and organizational level, various elements have been linked to WPV in general. Our study found that working part-time was a protective factor against WPV, consistent with existing evidence.^9^ Organizational measures such as training in WPV were associated with PV and PVV. The higher reported rates may reflect increased awareness of violence and a greater likelihood of a positive survey response.^11^ Notably, in our study, respondents were asked about general violence training, not specifically SV training. SV may be more challenging for women to identify and report due to passive reactions or shame,^38^ potentially explaining the positive connection between general violence training and PV or PVV, but not SV. Another factor related to PV and PVV was the ownership of the center. Public ownership of the center was identified as a protective factor, potentially linked to the presence of more protective laws and prevention plans in each autonomous community.^39^

Despite the similarities between PV and PVV, differences emerged when examining rural and urban center locations. PV was associated with mental health settings,^9^ older age, and dementia.^40^ The phenomenon of population aging, particularly pronounced in rural areas,^41^ may be connected to a higher prevalence of PV in these regions compared with urban areas. Existing studies exploring clinic location have reported mixed findings, potentially due to the lack of separate consideration for different types of violence or the need for more information on population characteristics. These aspects warrant further investigation to better understand the geospatial relationship with WPV for informed prevention measures.^25^ Concerning types of facilities, a distinct pattern emerged depending on the type of violence. Working in primary care appeared to provide a more secure practice setting for avoiding SV and PV, but not PVV. Other studies have indicated that clinicians working in emergency departments and similar facilities reported higher levels of violence compared with those in primary care and other settings, although this is subject to variations when multiple factors are considered together.^9^ Working in private clinics significantly increased the risk of experiencing SV, more than tripling it in comparison with primary care workers in our current study. This information is of particular relevance, given that a substantial proportion of physical therapists work in clinic or independent practice settings.^42^

Regarding treatment settings, it was found that therapy conducted in closed areas was associated with a higher risk of experiencing SV. In contrast, working in open areas reduced the likelihood of suffering SV, when compared with sessions conducted in areas with a mix of open and closed spaces. This connection between SV and closed treatment areas may be attributed to sexualization present in some related therapies.^18^^,^^43^ Some techniques involving physical contact may be misinterpreted^18^ and can be used as a pretext to justify sexual harassment by patients,^44^ especially when the treatment is administered in small or enclosed spaces.^19^

Additionally, although the current study identifies working with PWCI as a risk factor for SV, PV, and PVV according to established evidence^9^^,^^11^^,^^25^ it is possible that a more significant influence on PV with less impact on SV may be associated with the normalization of violence within specific populations such as PWCI or the elderly, as part of the work of health professionals.^45^ This observation also helps explain the higher rates of PV in mental health settings.^12^

It is important to note that there is no previous national study on type II WPV providing information about Spanish private and public physical therapists and their activities. Therefore, the results cannot be compared to assess their generalizability. According to data from the National Statistics Institute in Spain, the number of physical therapists in December 2021 was 62 691. We estimate the response rate to be over 5%, considering that this number includes all registered therapists, not just those actively working in the clinical area. It is possible that new practitioners decreased in 2021 compared with other years due to the COVID-19 pandemic.

### 4.1. Limitations

The present study has several limitations. Firstly, it used a cross-sectional design and an electronic survey aimed at the entire population of physical therapists with clinical experience. The reliance on self-reporting and clinician memory represents an inherent limitation of this research approach. In addition, it is not possible to establish causal relationships between the various personal, clinical, and professional factors analyzed and occurrence of any type of violence. Secondly, the survey items were developed specifically for this study, based on a prior review of the literature. Although the items were not subjected to formal psychometric testing, they were validated by field experts and pilot-tested on a small sample of physical therapists with diverse characteristics, including sex, age, clinical experience, and clinical settings. Future research could benefit from incorporating qualitative approaches to better understand how individuals perceive violence. This would lead to more precise definitions of type II WPV and related questions, thereby strengthening the evidence. Thirdly, the authors were encouraged by their university to use a specific survey program. However, due to inherent data confidentiality agreement restrictions, the program limited the types of questions and responses in the survey (eg, linking an open question to a closed answer “other”). Finally, the generalizability of the study findings may be somewhat limited as the sample may not be representative of all physical therapists. Due to the voluntary nature of the survey, individuals with a higher interest in the topic might have been more likely to complete it, potentially resulting in an overestimation of violence rates. Conversely, professionals who have experienced more severe forms of aggression might be hesitant or sensitive to the topic, leading them to avoid participation in the survey. This could result in an underestimation of the prevalence of violence.

## 5. Conclusions

The exposure to violence among physical therapists in Spain during their clinical practice falls within the lower to medium range of global prevalence, particularly when considering SV. PV is the least common type, whereas PVV and SV have similar occurrence rates.

The prevalence of violence is influenced by gender, with women and nonheterosexual clinicians being more susceptible to SV, whereas men are more likely to encounter PV.

Working fewer hours is associated with a lower incidence of any type of violence. Conversely, working with PWCI increases the risk, especially in the case of PV and PVV. The practice setting is a significant factor related to SV, with primary care being the most secure workplace, whereas private clinics pose the greatest risk for SV. Inpatient settings are most commonly associated with PV, whereas primary care centers are most linked to PVV.

Further research is required to assess the prevalence and types of violence experienced by physical therapists in their clinical roles, especially in Latin American regions where fewer studies have been conducted.

## Supplementary Material

Web_Material_uiae013
