# Supplementary material for: Prevalence and associated factors of sexual, psychological, and physical violence among physical therapists in their clinical role in Spain: a national web-based cross-sectional survey
Source: J Occup Health. 2024 Apr 11;66(1):uiae013. doi: 10.1093/joccuh/uiae013 (PMC11131336; doi:10.1093/joccuh/uiae013)
Supplement: Web_Material_uiae013 [file web_material_uiae013.zip › Table S1.docx]

Table S1. The percentage of variability explained by the model on the response (Pseudo R^2^) rises for the three types of violence ^a^

|  | SV | | PV | | PVV | |
| --- | --- | --- | --- | --- | --- | --- |
|  | GAM | GAM.W | GAM | GAM.W | GAM | GAM.W |
| Sensitivity | 1.71 | 71.71 | 0 | 76.64 | 1.23 | 67.81 |
| Specificity | 99.60 | 67.79 | 100 | 73.99 | 99.95 | 64.64 |
| AUC | 50.66 | 69.75 | 50 | 75.32 | 50.59 | 66.23 |
| Pseudo R^2^ | 9.85 | 16.70 | 9.98 | 22.81 | 7.89 | 11.23 |
